# Extraction, Enzymatic Modification, and Anti-Cancer Potential of an Alternative Plant-Based Protein from *Wolffia globosa*

**DOI:** 10.3390/foods12203815

**Published:** 2023-10-18

**Authors:** Warin Siriwat, Sunisa Ungwiwatkul, Kridsada Unban, Thunnop Laokuldilok, Warinporn Klunklin, Pipat Tangjaidee, Saranyapin Potikanond, Lovedeep Kaur, Suphat Phongthai

**Affiliations:** 1 Faculty of Agro-Industry, Chiang Mai University, Chiang Mai 50100, Thailand; ing.warin2@gmail.com (W.S.); kridsada.u@cmu.ac.th (K.U.); thunnop.l@cmu.ac.th (T.L.); wklunklin@gmail.com (W.K.); pipat.t@cmu.ac.th (P.T.); 2 Chemical Industrial Process and Environment Program, Faculty of Science, Energy and Environment, King Mongkut’s University of Technology North Bangkok (Rayong Campus), Rayong 21120, Thailand; sunisa.b@sciee.kmutnb.ac.th; 3 Center of Excellence in Agro Bio-Circular-Green Industry (Agro BCG), Faculty of Agro-Industry, Chiang Mai University, Chiang Mai 50100, Thailand; 4 Department of Pharmacology, Faculty of Medicine, Chiang Mai University, Chiang Mai 50200, Thailand; saranyapin.p@cmu.ac.th; 5 School of Food and Advanced Technology, Massey University, Palmerston North 4442, New Zealand; l.kaur@massey.ac.nz

**Keywords:** *Wolffia globosa*, alternative protein, protein hydrolysates, bioactive peptides, functional properties

## Abstract

The global plant-based protein demand is rapidly expanding in line with the increase in the world’s population. In this study, ultrasonic-assisted extraction (UAE) was applied to extract protein from *Wolffia globosa* as an alternative source. Enzymatic hydrolysis was used to modify the protein properties for extended use as a functional ingredient. The successful optimal conditions for protein extraction included a liquid to solid ratio of 30 mL/g, 25 min of extraction time, and a 78% sonication amplitude, providing a higher protein extraction yield than alkaline extraction by about 2.17-fold. The derived protein was rich in essential amino acids, including leucine, valine, and phenylalanine. Protamex and Alcalase were used to prepare protein hydrolysates with different degrees of hydrolysis, producing protein fragments with molecular weights ranging between <10 and 61.5 kDa. Enzymatic hydrolysis caused the secondary structural transformations of proteins from β-sheets and random coils to α-helix and β-turn structures. Moreover, it influenced the protein functional properties, particularly enhancing the protein solubility and emulsifying activity. Partial hydrolysis (DH3%) improved the foaming properties of proteins; meanwhile, an excess hydrolysis degree reduced the emulsifying stability and oil-binding capacity. The produced protein hydrolysates showed potential as anti-cancer peptides on human ovarian cancer cell lines.

## 1. Introduction

Although animal protein accounts for approximately 45 percent of the total human protein intake currently [1,2], animal protein consumption is tending towards decline due to environmental and social impacts, e.g., higher levels of greenhouse gas emissions per gram than soybean protein [3]. However, the traditional production of soybean protein to meet the global protein demand has been very challenging due to an insufficient supply of soybeans as a result of limited resources, particularly farmland [4], and the projected 50% increase in the world’s population [5]. Thus, alternative plant-based proteins are gaining more attention from food manufacturers and consumers worldwide.

Several researchers have reported the potential of using many plant materials or even by-products from agricultural processing as sources of alternative proteins, for example, lupin (*Lupinus angustifolius*) [6], quinoa (*Chenopodium quinoa*) [7], flaxseed (*Linum usitatissimum*) [8], peanut (*Arachis hypogaea*) [9], and rapeseed (*Brassica napus*) [10]. However, many challenges, including operation cost, extraction efficiency, acceptance by consumers, and the sustainability of those raw materials, should be considered when making decisions on commercializing these alternative proteins.

*Wolffia globosa* is an edible aquatic plant that has long been consumed throughout Thailand and its neighboring countries. It is recognized as a nutrient-rich plant, and is specifically composed of 34–45% protein, with essential amino acids including histidine, isoleucine, leucine, lysine, methionine, phenylalanine, threonine, valine, and tryptophan [11]. Generally, *Wolffia globosa* grows rapidly due to its high nutrient uptake efficiency, resulting in a short harvesting time of 1–2 weeks [12]. Moreover, it can be simply produced by being planted in a basin without competing for agricultural land [13]. Although it has great potential for being used as an alternative protein source, limited research has been conducted on the extraction of protein from *Wolffia globosa* and its utilization for functional food ingredients.

Recently, advanced technologies have been studied to extract many bioactive compounds from plant materials in order to overcome some of the limitations of the conventional methods, as well as to achieve a reasonable extraction yield. Protein is a target compound that has gained much attention from researchers worldwide. The application of ultrasonication based on the “cavitation effect” has been recognized as an efficient assisted method that successfully enhances the extraction yield of many researched crops, such as the extraction of hemicellulose from buckwheat hulls [14], oil from tobacco seed [15], polysaccharides from okra [16], and phenolics from green tea [17]. The mechanism of cavitation, including the formation and collapse of cavitation bubbles, destroys the cell wall of raw materials, allowing the release of protein to the solvent used through a higher rate of mass transfer. Due to its high efficiency, short operation time, and well-known advantage as a green technology, ultrasonic-assisted extraction might become a possible process for scaled-up protein production. Besides their nutritive values, proteins also have a variety of functional properties, such as foaming ability, emulsion ability, solubility, and oil absorption ability. These functional properties can also be modified via enzymatic hydrolysis. It has been reported that the degree of hydrolysis directly impacts the properties of protein. Partial hydrolysis can improve the foaming and emulsifying properties of protein, while excess hydrolysis decreases these properties [18,19]. In addition, enzymatic hydrolysis using different enzymes such as papain, Alcalase, Flavourzyme, Neutrase, and trypsin, is efficient in producing bioactive peptides without compromising nutritional value, with properties such as antioxidant activity [20], the inhibition of lipid peroxidation [21], angiotensin-converting enzyme [22], and anti-cancer [23]. The functional properties and bioactivities of proteins may vary according to the protein sources, amino acid profiles, and the types of enzymes used [24].

The objectives of this research were to study the feasibility of using ultrasonic-assisted extraction to produce an alternative protein from *Wolffia globosa*, as well as the modification of the derived protein using enzymatic hydrolysis. Moreover, the functional properties and bioactivities of protein hydrolysates were evaluated.

## 2. Materials and Methods

### 2.1. Materials and Chemical

*Wolffia globosa* was purchased from a commercial organic farm located in San Pa Tong, Chiang Mai, Thailand. Alcalase 2.4 L (2.4 AU/g) was purchased from Novozymes Co., Ltd., (Bangkok, Thailand). Protamex (1.5 MG) was donated by Brenntag Co., Ltd., (Bangkok, Thailand).

### 2.2. Raw Material Preparation

*Wolffia globosa* was dried in a hot air oven (Kluynamthai, Thailand) at 60 °C until the moisture content was below 10%, then mashed and sieved with a 40-mesh sieve. The sample powder (42.66 ± 0.22% carbohydrate, 23.68 ± 0.09% protein, 23.55 ± 0.03% ash, 8.01 ± 0.03% moisture content, and 2.11 ± 0.16% fat) was stored in a plastic zip-lock bag at −18 °C until use.

### 2.3. Alkaline Extraction (ALK)

*Wolffia globosa* powder was mixed with distilled water (1:30 *w*/*v*) and then its pH was adjusted to 10. The sample was mixed for 30 min with a magnetic stirrer. After centrifuging at 6000× *g* at 4 °C for 15 min, the top fraction was collected and the pH was adjusted to 3. The protein precipitate was collected via centrifugation under the same condition. The accumulated protein was pH-adjusted to 7 and freeze-dried (LABCONCO, Kansas City, MO, USA). The obtained powder was referred to as the “Protein concentrate: PC”.

### 2.4. Ultrasonic-Assisted Extraction (UAE)

Stat-Ease software (Design-Expert 6.0.2) was used for the experimental design and statistical analysis. The effects of three independent variables, including the liquid–solid ratio (X_1_, 30–50 mL/g), extraction time (X_2_, 10–30 min), and amplitude (X_3_, 60–80%), were evaluated using the response surface methodology (RSM) and a three-factor Box–Behnken design. The ultrasonic generator (VCX500, Sonics & Materials, Kansas City, MO, USA) was used to extract protein. The slurry of each treatment was further subjected to the protein precipitation step, as explained in the ALK section. The protein yield was calculated using the following equation:

Protein yield (%) = (protein in obtained/protein in raw material used) × 100(1)

The experimental data were fitted to a quadratic polynomial model, and a regression coefficient was obtained. The generalized quadratic model used in the response surface analysis is as follows:

(2) $$Y=\beta_{0}+\sum_{i=1}^{3}\beta iXi+\sum_{i=1}^{3}\beta ii{Xi}^{2}+\sum_{i=1}^{2}\sum_{i=2}^{3}\beta ijXiXj$$

where β_0_ is the constant, βi is the linear coefficient, βii is the quadratic coefficient, and βij is the interaction coefficient. Xi and Xj are the levels of the independent variables.

The effect and regression coefficients of individual linear, quadratic, and interaction terms were determined and generated in the analysis of variance (ANOVA) table. The significance of all terms in the polynomial was evaluated statistically by computing the F-value at a probability (P) of 0.05. The three-dimensional contour plots from the regression models were produced using statistical computations based on the regression coefficients.

### 2.5. Amino Acid Profiles

AOAC Official Method 994.12 (2000) [25] was used to analyze the amino acid profiles. The sample was dissolved in 6M HCl and then incubated at 110 °C for 24 h. Sodium citrate buffer was used to dilute the mixture and the pH was adjusted to 2.2. Individual amino acid components were separated and identified on a Zebron ZB-AAA GC column (10 mm × 0.25 mm, 0.25 µm film thickness) using gas chromatography (6890N; Agilent Technologies, Santa Clara, CA, USA) equipped with a transmission quadrupole mass spectrometer (5973 inert; Agilent Technologies). Norleucine was used as the internal standard. The content of each amino acid was expressed as mg/100 g protein.

### 2.6. Protein Pattern via Gel Electrophoresis

The protein pattern was examined using sodium dodecyl sulfate–polyacrylamide gel electrophoresis (SDS-PAGE) using the method described by Laemmli [26]. The sample with buffer (0.125 M Tris-HCl, pH 6.8, 4% SDS and 20% glycerol) was loaded onto 12% separation gel and 4% stacking. The sample was subjected to electrophoresis at a current of 15 mA/gel, then the gel was stained overnight with a 0.02% (*w*/*v*) Coomassie Brilliant Blue R-250 in 50% (*v*/*v*) methanol and 7.5% acetic acid for 40 min. After that, it was de-stained with 5% (*v*/*v*) methanol containing 7.5% (*v*/*v*) acetic acid for 20 min, then washed and dried.

### 2.7. Monitoring of Protein Secondary Structure Changes

The Fourier transform infrared spectrometer (FTIR) (Tensor 27, Bruker, Ettlingen, Germany) was used to measure the transmission infrared spectra of the protein samples. The spectra in the amide I region between 1600 and 1700 cm^−1^ were separated into multi-component peaks using the OriginPro 2022 program (OriginLab Corporation, Northampton, MA, USA), as described in Wang et al. [27].

### 2.8. Protein Hydrolysate (PH) Preparation

The protein concentrate was hydrolyzed with potent commercial enzymes, including Alcalase (0.0888 unit) and Protamex (0.0272 unit), at the optimal conditions (55 °C and pH 8.5; 50 °C and pH 7.0), according to the method of Phongthai et al. [28]. Each enzyme at a ratio of 100:1 (*w*/*w*) for Alcalase and 100:10 (*w*/*w*) for Protamex was mixed with the sample. The mixture was hydrolyzed in optimal conditions. Then, the mixture’s pH was adjusted to 4 to terminate the enzyme activity. The pH was then adjusted to 7 before centrifuging at 2200× *g* at 4 °C for 10 min. The top fraction was collected and freeze-dried. The degree of hydrolysis was determined using the pH-stat method, and the following formula was used:

DH = (BN_b_/αh_tot_M_p_) × 100 (3)

where B is the volume of NaOH used (mL); N_b_ is the normality of the NaOH; M_p_ is the mass of the protein in g (N × 6.25); h_tot_ is the total number of peptide binds in the protein substrate (7.40 meq/g protein); and α is the average degree of dissociation of the α-NH_2_ groups.

### 2.9. Functional Property Determinations

#### 2.9.1. Solubility

The method of Xia et al. [29] was used to determine the protein solubility. The protein samples were mixed with distilled water (1% *w*/*v*) and pH-adjusted to 3, 5, 7, 9, and 11. The samples were mixed using magnetic stirring for 30 min, then centrifuged at 5500 rpm for 15 min. The Bradford method was used to determine the supernatant’s protein content. The standard was Bovine serum albumin (BSA). The following equation was used to compute the protein solubility:

Solubility (%) = (Protein content in supernatant/Total protein content) × 100(4)

#### 2.9.2. Emulsifying Properties

The emulsifying properties were analyzed using the method of Phongthai et al. [28]. The protein sample solutions (5% *w*/*v*, 10 mL) were mixed with 10 mL of soybean oil and homogenized at 10,000 rpm for 1 min. The following equation was used to calculate the emulsifying properties:

Emulsifying activity (%) = (A/B) × 100(5)

Emulsifying stability (%) = (A_incubate_/B) × 100 (6)

where A is the volume of the emulsifying layer (mL) after centrifugation, B is the total volume (mL), and A_incubate_ is the volume of the emulsifying layer (mL) remained after incubating at 80 °C for 10 min.

#### 2.9.3. Foaming Properties

The method of Phongthai et al. [28] was used to determine the foaming properties. The protein samples were mixed with distilled water (1% *w*/*v*, 20 mL) and homogenized at 10,000 rpm for 1 min. After whipping, the total volume was measured after 0 and 30 min. The following equation was used to calculate the foaming properties:

Foaming activity (%) = (A − B)/B ×100(7)

Foam stability (%) = (A_30min_ − B)/(A_0min_ − B) ×100 (8)

where A is the volume after whipping (mL) and B is the volume before whipping (mL).

#### 2.9.4. Oil-Binding Capacity

The oil-binding capacity was determined by modifying the method of Cao et al. [30]. The protein samples (0.5 g) were mixed with soybean oil (5.0 mL) and centrifuged at 1200× *g* for 15 min. The top fraction was drained, and the volume of gain was measured as the oil-binding capacity. The following equation was used to calculate the oil-binding capacity:

Oil-binding capacity = sample with oil absorbed (g)/sample (g)(9)

### 2.10. Cell Viability Assay

The cytotoxic activity of the protein hydrolysates on a normal cell line (LX2) and a human ovarian cancer cell line (A2780) using an MTT assay was investigated, following the method of Paramee et al. [31]. The cells were seeded at a density of 1 × 10^4^ cells per well in 96-well plates overnight and treated with protein hydrolysates in quadruplicate. For the treatment group, the cells were incubated with complete media containing different concentrations of protein samples, ranging from 0 to 1 mg/mL. After 24 h, the cells were incubated with 0.5 mg/mL of the MTT reagent (Applichem GmbH, Germany) for 1–3 h. The culture supernatant was aspirated, and 100 μL of DMSO was added to each well. The absorbance was measured at 570 nm using a Synergy™ H4 Hybrid Multi-Mode Microplate Reader. The cell viability assay was performed over 3 individual experiments.

### 2.11. Statical Analysis

All experiments are expressed as the mean ± SD in triplicate. The results were analyzed using a one-way analysis of variance (ANOVA). The means were compared with the Duncan test (*p* < 0.05) using the SPSS Statistics program (Version 17.0).

## 3. Results and Discussion

### 3.1. Model Fitting and Statistical Analysis

The protein extraction yields derived from 17 combination treatments among the studied variables are listed in Table 1. The analysis of variance for the response surface quadratic model is reported in Table 2. The model was significantly (*p* < 0.05) fitted, with a model *F*-value of 620.54. The linear terms (X_1_, X_2_, X_3_) and quadratic term of the liquid–solid ratio (X_1_^2^) significantly impacted the response value. However, the interaction terms of the liquid–solid ratio, extraction time, and amplitude were not significant. The determination coefficient (R^2^) value of the extraction yield was 0.9087, which mean that 90.87% of the variation in the response value can be explained by the fitted model. Meanwhile, the lack of fit value was insignificant, with a value of 0.7748 (*p* > 0.05), demonstrating that the model adequately represent the real relationships among the parameters chosen. The regression equation for the extraction yield was given as follows:

Y = 9.37 − 5.26X_1_ + 0.28X_2_ + 0.27X_3_  + 1.56X_1_^2^ (10)

where X_1_ is the liquid–solid ratio (mL/g), X_2_ is the extraction time (min), and X_3_ is the amplitude (%).

### 3.2. Influence of Independent Factors on the Protein Extraction Yield

**Figure 1:**
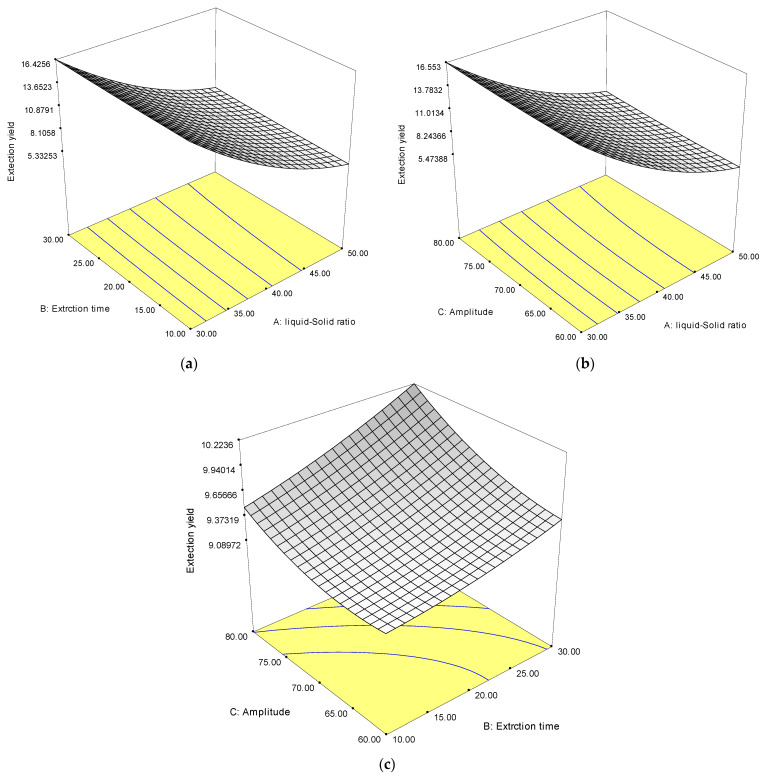
The response surface plots of independent variables on the protein extraction yield (%), extraction time and liquid-solid ratio (**a**), amplitude and liquid-solid ratio (**b**), and amplitude and extraction time (**c**).

The response surface plots of the protein extraction are shown in Figure 1. The effects of the liquid–solid ratio and extraction time on the protein extraction yield are shown in Figure 1a, while amplitude was fixed at 70%. The result indicates that the protein extraction yield increased as the extraction time increased. The highest yields were obtained with an extraction time of 30 min and a solid–liquid ratio of 30 mL/g. A similar result regarding the improvement of protein extraction from brewer’s spent grain using an ultrasonicator was reported by Tang et al. [32]. This may be explained by the fact that a lower liquid–solid ratio increases the extraction productivity by producing a difference concentration between the inside cell of *Wolffia globosa* and the outside alkaline solvent; this eventually increases the mass transfer rate of soluble proteins, resulting in an increased extraction yield [33].

Figure 1b shows the extraction yield with varying amplitudes and liquid–solid ratios at a fixed extraction of 20 min. It was observed that the *Wolffia globosa* protein extraction yield clearly increased as the amplitude increased and the liquid–solid ratio decreased. This may be attributed to the ultrasonic mass transfer principle and energy dissipation in the solution during extraction [28].

Figure 1c shows the extraction yield with varying amplitudes and extraction times at a fixed liquid–solid ratio of 30 mL/g. The extraction yield increased with the increasing amplitudes and extraction times. This might be explained by the creation of microbubbles during the sonication process, which aids in protein extraction from *Wolffia globosa* [34].

### 3.3. Optimization and Validation

The protein yields based on alkaline and UAE extraction are compared and listed in Table 1. The experimental response values of each treatment was very close to the predicted values, supporting the suitability of the generated model equation. The protein yield of the UAE-optimal condition (14.13 ± 1.20%) was higher than that of alkaline extraction (6.77 ± 0.53%) by about 2.09-fold, due to the fact that the disruption and disintegration of plant cells can be facilitated by cavitation phenomena, which enhance the penetration of a solution into interior structures, increasing the extraction yield. The protein concentrate contained 51.33% of the protein content. Therefore, the UAE-optimal conditions of a 30 mL/g liquid–solid ratio, 25 min of extraction time, and 78% amplitude were selected to prepare *Wolffia globosa* protein concentrates for the next experiment.

### 3.4. Amino Acid Profiles

The amino acid profiles of protein concentrate are displayed in Table 3. The derived protein was rich in essential amino acids, including leucine (3284.99 ± 5.11 mg/100 g sample), valine (2459.10 ± 9.82 mg/100 g sample), and phenylalanine (1952.33 ± 6.93 mg/100 g sample). In general, hydrophobic amino acids including leucine or valine, as well as aromatic amino acids (phenylalanine, tryptophan, and tyrosine), have a strong antioxidant activity due to their electron- or hydrogen-donating ability [34]. In addition, hydrophobic amino acids play a key role in functional properties, particularly the oil-binding and emulsifying properties [35]. In addition, the total essential amino acids of this protein concentrate were 44.90%, which was less the total essential amino acids found in soybeans [36]. However, the *Wolffia globosa* protein concentrates contained higher amounts of total essential amino acids than the daily required intakes suggested by the FAO/WHO, especially regarding their leucine and valine contents. The data suggested that *Wolffia globosa* protein concentrate is a suitable source of protein nutrition for human consumption, as well as a potential raw material for the production of functional ingredients.

### 3.5. Protein Hydrolysis and Protein Patterns

As regards the protein hydrolysis patterns, Protamex seemed to have higher efficiency in the hydrolysis of *Wolffia globosa* protein than Alcalase at the beginning stage, as a shorter time was taken for the hydrolysis. However, the hydrolysis efficiency of Protamex was lower than that of Alcalase, as the hydrolysis time was extended. The protein hydrolysates produced using Protamex with different DH values of 3% (PDH3%), 6% (PDH6%), and 9% (PDH9%) were obtained at the hydrolysis times of 2.62, 21.31, and 86.75 min, respectively, whereas the protein hydrolysates prepared with Alcalase, with different DH values of 3% (ADH3%), 6% (ADH6%), and 9% (ADH9%), were obtained at the hydrolysis times of 5.81, 18.18, and 39.14 min, respectively. This is because Protamex is an endo-protease with a broad specificity to hydrophobic amino acids; this substrate decreased as the hydrolysis time increased, and thus the hydrolysis rate was accordingly reduced. Meanwhile, Alcalase is an endo-protease of the serine type with a very broad substrate specificity that can hydrolyze most peptide bonds within a protein molecule. As a result, the different type of enzyme exerts a specific enzyme activity; thus, it directly influenced the degree of hydrolysis. However, a low ratio of enzyme-to-protein substrate can also limit the degree of hydrolysis; therefore, the use of a higher ratio of enzymes could enhance the degree of hydrolysis [37]. 

**Figure 2:**
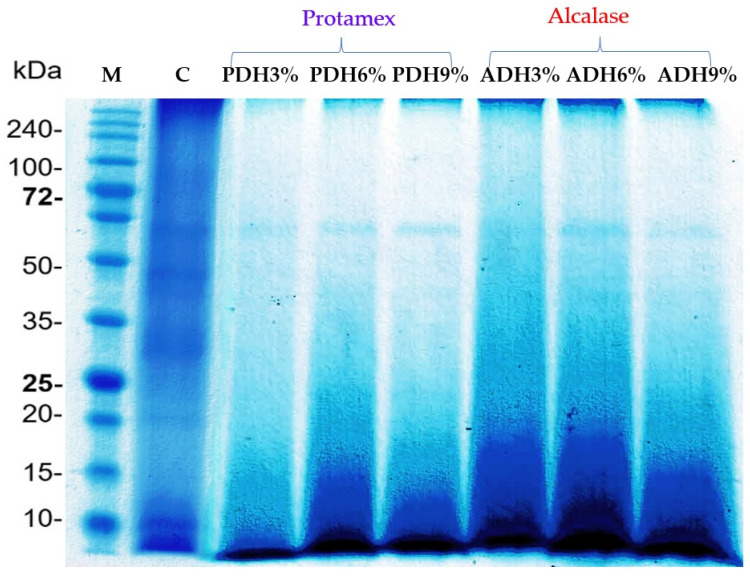
Protein pattern of protein concentrate and hydrolysates prepared with Protamex and Alcalase at DH values of 3%, 6%, and 9%.

The protein patterns of the *Wolffia globosa* protein concentrate and the hydrolysates prepared using Protamex and Alcalase at DH values of 3%, 6%, and 9% are shown in Figure 2. The SDS-PAGE showed the five main protein bands in the non-hydrolyzed sample at 20.0, 25.0, 30.6, 45.6, and 61.5 kDa. This result was consistent with the study of Duangjarus et al. [38], who reported that *Wolffia globosa* protein bands were identified at 25, 45, 50, and 63 kDa. After hydrolysis, the intensity of these protein bands decreased; meanwhile, new, smaller bands clearly appeared at the bottom of the gel. This result confirmed that enzymatic hydrolysis affected the molecular weight (MW) distribution of the protein [39]. The protein bands with MWs of 20.0, 25.0, 30.6, and 45.6 kDa were completely hydrolyzed with Protamex and Alcalase; meanwhile, the protein band with a MW of 61.5 kDa was very stable against digestion by the enzymes used. This is possibly due to the lack of substrates in the protein structure and/or its ability to re-fold to its native structure, even when subjected to enzymatic hydrolysis.

### 3.6. Changes of Secondary Structures of Proteins

The secondary structures of the proteins were analyzed using FTIR spectroscopy. The Gaussian model was selected for multi-component division. The infrared spectra of the proteins showed a variety of amide bands that correspond to various peptide moiety vibrations. The amide I in the range of 1600–1700 cm^−1^ was the most widely studied range of the peptide group for studying the secondary structures of proteins [40]. This region was divided into four ranges for α-helix (1650–1660 cm^−1^), β-sheet (1600–1640 cm^−1^), β-turn (1660–1700 cm^−1^), and random coil (1640–1650 cm^−1^) structures [41]. The portion percentage of each component was determined using the peak areas, as concluded in Table 4.

Apparently, enzymatic hydrolysis affected the secondary structure changes in the *Wolffia globosa* protein (Table 4). The non-hydrolyzed protein consisted of 26.55% α-helix, 18.45% β-sheet, 7.94% β-turn, and 47.06% random coil structures. After enzymatic hydrolysis, the α-helix and β-turn structures greatly increased. Meanwhile, the portions of β-sheets and random coils were reduced. This result suggests that hydrolysis using these two enzymes can convert the β-sheets and random coils to α-helix and β-turn structures. Similarly, Akbari et al. [42] reported that the content of α-helix and β-turn structures in potato protein hydrolysates increased, while the β-sheets decreased after enzymatic hydrolysis. This change might improve the emulsifying activity of the protein due to the amphipathic structure of α-helices and the greater surface hydrophobicity of β-turn structures. However, the amino acid composition, type of enzyme used, and inter- and intra-molecular interactions in protein structures may influence the modification of the proteins’ secondary structures in different ways [43].

### 3.7. Functional Properties

#### 3.7.1. Solubility

The solubility of the *Wolffia globosa* protein concentrate and hydrolysates prepared using Alcalase and Protamex is summarized in Table 4. The protein solubility of the protein concentrate and hydrolysates increased as a function of the pH. All samples displayed the lowest levels of solubility at pH 3, which is close to the protein’s isoelectric point, where the protein’s net charge is zero, resulting in the precipitation of proteins. As a result, the protein hydrolysate samples were more soluble than the protein concentrate. The protein hydrolysate at a DH of 9% showed the highest solubility (85.1 + 4.14%) at pH 11, followed by the protein hydrolysates with a DH of 6% and a DH of 3%, respectively. Similarly, Chabanon et al. [44] reported that increasing the hydrolysis degree enhanced the solubility of the globulin form of rapeseed protein isolates. Barac et al. [45] reported that a pea (*Pisum sativum* L.) protein isolate was more soluble after enzymatic hydrolysis using papain. In addition, rice bran protein hydrolysates with a DH of 15.04% and cowpea protein hydrolysates with a DH of 20% had the highest solubility when compared to the samples with lower hydrolysis degrees and non-hydrolyzed proteins [28,45]. The enhanced solubility of the protein hydrolysates may be explained by the enzymatic hydrolysis having produced smaller peptides, which are more polar than the polypeptides in the protein concentrate; thus, they are more soluble in aqueous solutions because they can form stronger hydrogen bonds with water [46].

#### 3.7.2. Foaming Properties

The foaming ability and foam stability of the *Wolffia globosa* protein concentrate and hydrolysates are presented in Table 4. A considerable improvement in the foaming ability was observed for the protein hydrolysates with a DH of 3% (55.00 ± 0.00% and 50.00 ± 0.00% for Alcalase and Flavourzyme hydrolysates, respectively), which were significantly higher than that of the protein concentrate (42.21 ± 1.12%, *p* < 0.05). Similarly, in our previous report [28], partial hydrolysis using Alcalase at a DH of 5.04% improved the foam activity of rice bran protein the most. However, the foaming ability tended to be significantly reduced as the DH increased. Kong et al. [47] also reported poorer foam properties of wheat gluten hydrolysates when the DH extremely increased. In terms of the foam stability, the protein hydrolysate with a low degree of hydrolysis promoted the highest foam stability (33.33 + 4.95%); however, it was not significantly different to the value of the protein concentrate (29.68 ± 0.56%, *p* > 0.05). Meanwhile, the increase in the DH to 6% or 9% negatively impacted the foam stability. As found by Bandyopadhyay et al. [48], the decreased foam stability was observed in sesame (*Sesamum indicum* L.) protein hydrolysates with high DH values. This might be due to excessive hydrolysis leading to the loss of native protein structures by creating small peptide fractions that have a poor ability to form film layers around gas bubbles [35]. The protein hydrolysates prepared using Alcalase were better at foam stabilizing than those prepared with Protamex. This may be due to the different enzymes having produced protein hydrolysates with varying peptide sizes and charges.

#### 3.7.3. Emulsifying Properties

The emulsifying activity and emulsifying stability of the *Wolffia globosa* protein concentrate and hydrolysates are listed in Table 4. The protein hydrolysates had a better emulsifying activity than the protein concentrate. As the compact tertiary structure of the native proteins is disrupted by hydrolysis, smaller peptides with an improved hydrophilic–hydrophobic balance may be generated. This could facilitate the diffusion and adsorption at the interface of the proteins, resulting in a higher emulsifying activity. In addition, the *Wolffia globosa* protein concentrate contained a high amount of hydrophobic amino acids, including leucine, valine, and phenylalanine. As the protein’s hydrophobic site was exposed, it led to an increased surface activity and increased adsorption at the interface, resulting in an increased emulsifying ability [49]. However, the emulsifying stability of the protein hydrolysates prepared with Alcalase and Protamex at the same hydrolysis degree were not significantly different (*p* > 0.05). The emulsifying stability of the protein hydrolysates decreased with the increasing DH, which corresponded to the study of Kong et al. [47] in which a degree of hydrolysis beyond 5% reduced the emulsifying stability of gluten hydrolysates. In addition, similar trends were found in peanut and rapeseed protein hydrolysates [44,50]. This might be due to the smaller peptides that are produced under higher hydrolysis degrees being unable to form elastic films around oil droplets as strongly as larger peptides can [35].

#### 3.7.4. Oil-Binding Capacity

The oil-binding capacity of the *Wolffia globosa* protein concentrate and hydrolysates is listed in Table 4. The protein concentrate had the highest oil-binding capacity (4.65 ± 0.05 g oil/g sample) among the studied samples. The oil-binding capacity is generally correlated with the hydrophobicity of proteins. *Wolffia globosa* protein contains a high content of hydrophobic amino acids, including leucine, valine, and phenylalanine, that play a major role in interactions between the protein and oils, resulting in an improved oil-binding capacity [51]. However, the oil-binding capacity of the protein hydrolysates decreased as the DH increased. The result was correlated to the study of Guan et al. [52], where extensive enzymatic hydrolysis notably diminished the oil-binding capacity of oat bran protein hydrolysates. During enzymatic hydrolysis, the peptide bonds in protein structures are broken, producing small fragments of peptides as well as exposing ionizable groups, which results in a decreased oil-binding capacity [53].

### 3.8. The Effect of Protein Hydrolysates on Cell Viability

**Figure 3:**
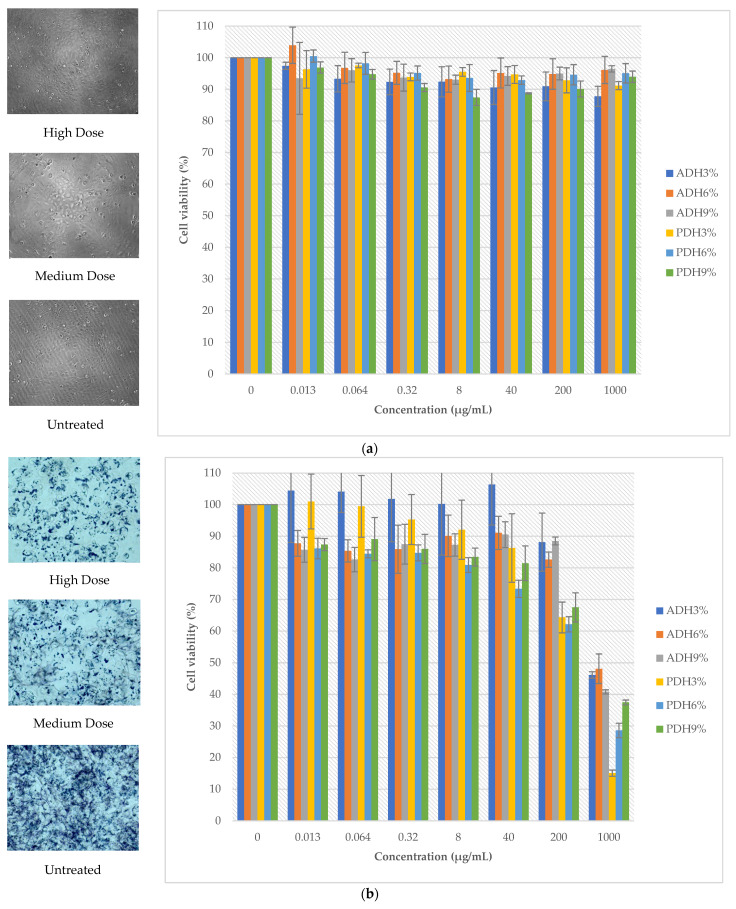
Cell viability of LX2 (**a**) and A2780 cell lines (**b**) treated with protein hydrolysates.

The cell viability of the LX2 and A2780 cell lines is shown in Figure 3. The protein hydrolysates prepared using Alcalase and Protamex showed no cytotoxicity to normal cells, since the percentage of cell viability was above 80% (Figure 3a) at all treated doses. Meanwhile, the protein hydrolysates, particularly PDH3%, PDH6%, and PDH9%, had a dose-dependent inhibitory effect on the growth of the cancer cell line: the A2780 cell line (Figure 3b). Clearly, the cell viability of the A2780 cell line was less than 50% at the maximum dose of the protein hydrolysates. The composition and amino acid sequence of the peptides were important factors for having an antiproliferative effect [54]. Moreover, the hydrophobicity of peptides is the main property that affects the cancer inhibition of peptides. The release of bioactive hydrophobic peptides of *Wolffia globosa* protein during enzymatic hydrolysis might be the cause of the inhibition of cancer cells, particularly the aromatic amino acid residues of bioactive hydrophobic peptides, including tyrosine, phenylalanine, and tryptophan, which can inhibit the growth of cancer cell lines by inducing apoptosis and suppressing the cell cycle [55]. Hydrophobic peptides can increase the interactions between anti-cancer peptides and the membrane bilayers on the outer leaflets of tumor cells [56]. Singh et al. [57] reported that the anti-cancer peptides from soybean containing > 60% of hydrophobic amino acids in their sequences (Phe-His-Pro-Phe-Pro-Arg and Asn-Trp-Phe-Pro-Leu-Pro-Arg) showed a significant inhibitory effect on the proliferation of colon cancer Caco-2 cells by inhibiting histone deacetylase 1 (HDAC1) activity and regulating the expression of the cancer-related genes. Therefore, purification and identification techniques, e.g., membrane ultrafiltration, column chromatography, and LC-MS/MS, should be further conducted in order to investigate the specific amino acid sequences in our *Wolffia globosa* peptides that are responsible for anti-cancer efficacy.

## 4. Conclusions

The application of a high-intensity ultrasound was successful for protein extraction from *Wolffia globosa*. The protein extraction yield of the optimal condition was higher than that of alkaline extraction by about 2.17-fold. The most significant extraction parameters included the liquid–solid ratio, extraction time, and amplitude. The derived protein concentrate contains a high content of essential amino acids, particularly leucine, valine, and phenylalanine. Enzymatic hydrolysis modified the secondary structures of proteins by transforming β-sheets and random coils to α-helices and β-turns. The hydrolysis enhanced the protein solubility and emulsifying activity; meanwhile, partial hydrolysis improved the foaming and emulsifying properties. The generated bioactive peptides showed potential for inhibiting the human ovarian cancer cell line; however, isolation and identification via the sequencing of peptides needs to be further conducted in order to reveal the specific peptides contributing to anti-cancer activity. Therefore, it can be concluded that *Wolffia globosa* protein is suitable to be counted as a nutritious alternative plant-based protein and to be used as a raw material for the production of novel functional ingredients.

## Figures and Tables

**Table 1:** Protein extraction yield of *Wolffia globosa* via UAE and alkaline extraction.

Run	Factors			Response Value	Predict Value
	Liquid–Solid Ratio (mL/g)	Time (min)	Amplitude (%)		
1	30	10	70	16.12	16.08
2	50	10	70	5.25	5.33
3	30	30	70	16.51	16.43
4	50	30	70	6.09	6.12
5	30	20	60	16.14	16.14
6	50	20	60	5.59	5.47
7	30	20	80	16.43	16.55
8	50	20	80	6.15	6.16
9	40	10	60	9.08	9.11
10	40	30	60	9.40	9.49
11	40	10	80	9.56	9.47
12	40	30	80	10.26	10.22
13	40	20	70	9.56	9.37
14	40	20	70	9.08	9.37
15	40	20	70	9.14	9.37
16	40	20	70	9.51	9.37
17	40	20	70	9.56	9.37
Optimal	30	25	78	14.13	15.70
Alkaline extraction (pH 10, stirring for 30 min)				6.52	-

**Table 2:** Analysis of variance for response surface quadratic model.

Source	Sum of Square	df	Mean of Square	*F*-Values	Prob > *F*
Model	233.69	9	25.97	620.54	0.0001
X_1_	221.75	1	221.75	5299.50	0.0001
X_2_	0.63	1	0.63	15.17	0.0059
X_3_	0.60	1	0.60	14.42	0.0067
X_1_^2^	10.28	1	10.28	245.75	0.0001
X_2_^2^	0.01	1	0.01	0.33	0.5862
X_3_^2^	0.09	1	0.09	2.15	0.1856
X_1_X_2_	0.05	1	0.05	1.18	0.3142
X_1_X_3_	0.02	1	0.02	0.42	0.5395
X_2_X_3_	0.04	1	0.04	0.84	0.3896
Lack of Fit	0.7748				
R^2^	0.9987				
Adj R^2^	0.9971				

**Table 3:** The essential amino acids of *Wolffia globosa* protein concentrate (mg/100 g sample).

Essential Amino Acids	*Wolffia globosa* Protein Concentrate	Soybean Protein Concentrate	FAO/WHO
Histidine	772.12 ± 13.02	1546	1600
Isoleucine	1357.53 ± 2.75	2402	1300
Leucine	3284.99 ± 5.11	3896	1900
Lysine	1584.08 ± 4.38	3306	1600
Methionine	464.01 ± 1.34	608	1700
Phenylalanine	1952.33 ± 6.93	2620	1900
Threonine	1205.05 ± 0.54	1910	900
Valine	2459.10 ± 9.82	2728	1300
Total	13,079.21	19,016	12,200

**Table 4:** Functional properties, antioxidant properties, and secondary structure portions of protein concentrate and hydrolysates.

Properties of Protein	Protein Concentrate	Alcalase			Protamex		
		DH 3%	DH 6%	DH 9%	DH 3%	DH 6%	DH 9%
Functional Properties							
Solubility							
pH 3	1.60 ± 0.90 ^Dd^	34.47 ± 4.57 ^Cd^	56.30 ± 3.14 ^Ac^	56.97 ± 3.15 ^Ab^	50.72 ± 1.42 ^Ab^	44.07 ± 0.45 ^Bd^	62.81 ± 2.97 ^Ab^
pH 5	25.76 ± 5.09 ^Cc^	57.76 ± 4.19 ^Bc^	67.92 ± 1.13 ^Ab^	70.08 ± 4.46 ^Ab^	61.61 ± 2.42 ^ABb^	63.01 ± 3.52 ^ABc^	64.81 ± 1.93 ^Ab^
pH 7	57.07 ± 6.01 ^Bb^	63.01 ± 4.86 ^Bbc^	76.06 ± 2.13 ^Aab^	80.48 ± 6.24 ^Aa^	63.22 ± 4.86 ^Bb^	63.46 ± 1.56 ^Bc^	65.61 ± 3.62 ^Bb^
pH 9	69.02 ± 8.22 ^Ba^	70.39 ± 5.26 ^ABab^	76.23 ± 4.10 ^ABab^	83.36 ± 7.72 ^Aa^	71.08 ± 2.98 ^ABa^	74.89 ± 2.23 ^ABb^	76.60 ± 2.12 ^ABa^
pH 11	79.70 ± 5.29 ^ABa^	71.54 ± 2.71 ^Ba^	83.39 ± 3.11 ^Aa^	85.12 ± 4.14 ^Aa^	76.43 ± 3.86 ^ABa^	83.76 ± 4.13 ^Aa^	80.60 ± 4.26 ^ABa^
Foaming activity (%)	42.21 ± 1.12 ^cd^	55.00 ± 0.00 ^a^	46.69 ± 5.77 ^bc^	46.67 ± 5.77 ^bc^	50.00 ± 0.00 ^ab^	35.00 ± 5.00 ^e^	36.67 ± 2.89 ^de^
Foam stability (%)	29.68 ± 0.56 ^ab^	33.33 ± 1.86 ^a^	28.33 ± 2.89 ^b^	27.14 ± 4.95 ^bc^	26.67 ± 0.00 ^bc^	23.43 ± 1.42 ^cd^	21.96 ± 0.46 ^d^
Emulsifying activity (%)	96.00 ± 3.40 ^b^	100.00 ± 0.00 ^a^	100.00 ± 0.00 ^a^	100.00 ± 0.00 ^a^	100.00 ± 0.00 ^a^	100.00 ± 0.00 ^a^	100.00 ± 0.00 ^a^
Emulsifying stability (%)	80.57 ± 0.49 ^a^	63.89 ± 1.20 ^ab^	62.5 ± 0.00 ^cd^	62.5 ± 0.00 ^cd^	65.28 ± 1.20 ^b^	62.50 ± 0.00 ^cd^	61.81 ± 1.20 ^d^
Oil-binding capacity (g/g)	4.65 ± 0.05 ^a^	2.23 ± 0.01 ^c^	2.21 ± 0.01 ^c^	1.83 ± 0.01 ^d^	3.51 ± 0.07 ^b^	2.23 ± 0.07 ^c^	1.86 ± 0.06 ^d^
Secondary Structure Portion (%)							
α-helix	26.55	29.71	40.26	37.64	36.70	34.05	29.56
β-sheet	18.45	13.93	13.64	13.63	12.79	13.25	14.03
β-turn	7.94	10.24	10.07	11.09	11.52	10.67	9.45
Random coils	47.06	46.13	36.03	37.64	38.98	42.02	46.95

Different lowercase letter in the same row and uppercase letter in the same column indicate significant differences (*p* < 0.05).

## Data Availability

Data is contained within the article.
